# Organizing the Present, Looking to the Future: An Online Knowledge Repository to Facilitate Collaboration

**DOI:** 10.2196/jmir.2.2.e10

**Published:** 2000-06-23

**Authors:** Charles Burchill, Leslie L Roos, Patricia Fergusson, Laurel Jebamani, Ken Turner, Stephen Dueck

**Affiliations:** ^1^Manitoba Centre for Health Policy and EvaluationDepartment of Community Health SciencesFaculty of MedicineUniversity of ManitobaSt. Boniface General Hospital Research CentreCanada

**Keywords:** Epidemiologic Methods, Concept Dictionary, Knowledge Bases (Computer), Internet, Cooperative Behavior, Administrative Data, Education, Research Support

## Abstract

**Background:**

Comprehensive data available in the Canadian province of Manitoba since 1970 have aided study of the interaction between population health, health care utilization, and structural features of the health care system. Given a complex linked database and many ongoing projects, better organization of available epidemiological, institutional, and technical information was needed.

**Objective:**

The Manitoba Centre for Health Policy and Evaluation wished to develop a knowledge repository to handle data, document research methods, and facilitate both internal communication and collaboration with other sites.

**Methods:**

This evolving knowledge repository consists of both public and internal (restricted access) pages on the World Wide Web (WWW). Information can be accessed using an indexed logical format or queried to allow entry at user-defined points. The main topics are: Concept Dictionary, Research Definitions, Meta-Index, and Glossary. The Concept Dictionary operationalizes concepts used in health research using administrative data, outlining the creation of complex variables. Research Definitions specify the codes for common surgical procedures, tests, and diagnoses. The Meta-Index organizes concepts and definitions according to the Medical Sub-Heading (MeSH) system developed by the National Library of Medicine. The Glossary facilitates navigation through the research terms and abbreviations in the knowledge repository. An Education Resources heading presents a web-based graduate course using substantial amounts of material in the Concept Dictionary, a lecture in the Epidemiology Supercourse, and material for Manitoba's Regional Health Authorities. Confidential information (including Data Dictionaries) is available on the Centre's internal website.

**Results:**

Use of the public pages has increased dramatically since January 1998, with almost 6,000 page hits from 250 different hosts in May 1999. More recently, the number of page hits has averaged around 4,000 per month, while the number of unique hosts has climbed to around 400.

**Conclusions:**

This knowledge repository promotes standardization and increases efficiency by placing concepts and associated programming in the Centre's collective memory. Collaboration and project management are facilitated.

## Introduction

Worldwide, an increasing number of research centers are using administrative databases for monitoring and evaluating health care systems. In several Canadian provinces, comprehensive health care data are made available for research not only by an insurance plan that covers almost the entire population, but also by a strong commitment to developing this resource. The Manitoba Centre for Health Policy and Evaluation (MCHPE), a university-based research unit, has been using the Manitoba Health database since 1991. MCHPE's founders began working with these data in 1975; the database has been developing incrementally since 1970.

**Figure 1:**
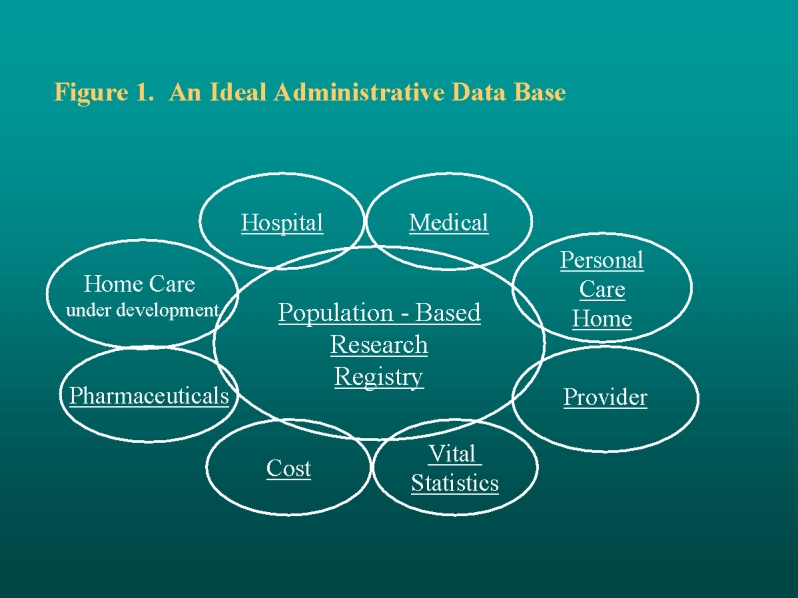
An Ideal Administrative Database

Manitoba's database documents almost every contact that the population has with the health care system. Figure 1 presents an idealized view of the MCHPE database with a population-based research registry playing a central role. The research registry contains a unique, encrypted number assigned to each provincial resident, together with information on demographic characteristics, location of residence, and family composition. The substantive files include information on hospital stays, physician visits, nursing home stays, pharmaceutical use, and so forth. These files use the same number to track utilization of services, making it possible to compile comprehensive histories for individuals over time. Similar databases outside Canada (Oxford Record Linkage Study, Scottish Record Linkage System, Rochester Epidemiology Project, Western Australian Health Services Research Linked Database) have recently been reviewed. The Manitoba database was noted as representing "international best practice" across a number of benchmarks [1]. Approximately 300 papers or reports have been written using the Manitoba data (MCHPE Papers Published).

With the advent of technologies facilitating linkages between databases, Manitoba researchers have been better able to study the complex relations between population health, health care utilization, and structural features of the health care system [2]. As part of its long-range plan, MCHPE hopes to expand its database by linking the existing health files to population-based data on students, families, and classrooms from Manitoba's Department of Education.

MCHPE has required an efficient system to manage this information resource, to document research methods, and to facilitate internal communication and collaboration with other sites. Within Winnipeg, MCHPE has staff members located at three different University of Manitoba campuses. Additional staff and active collaborators are distributed throughout North America. As much as possible, we wish to facilitate work and interaction as if the researchers were in the same physical space [3].

Over nine years of operation, MCHPE has amassed a vast amount of epidemiological, institutional, and technical information. It can be a formidable task to identify the local expert to assist with a particular topic or to access up-to-date material on orientation; research projects and protocols; data dictionaries; and guidelines for security, access, and confidentiality. How do we update documentation and ensure consistency to permit replication and extension of studies? How do we prevent re-creating the wheel after other investigators have already operationalized research concepts? This paper describes the development, current status, and future directions of the "repository of shared knowledge" at MCHPE [3]. We have been guided not only by the need for efficient data management and standardized research methods within MCHPE, but also by the wish to make the "Centre's intellectual assets more widely available to scholars, students and other health care researchers" [4]. Our long-term goal is to produce a knowledge system that incorporates concepts and methods from other sources, documenting alternative perspectives and mentioning shortcomings or potential problems.

## Methods

### Knowledge Repository

The MCHPE knowledge repository is an evolving enterprise, consisting of both external (i.e., public) and internal Web pages [5]. External users can access the public website available over the Internet as part of the MCHPE home page on the World Wide Web (WWW) (URL: http://www.umanitoba.ca/centres/mchpe). The internal pages are accessible only by authorized individuals.

**Figure 11:**
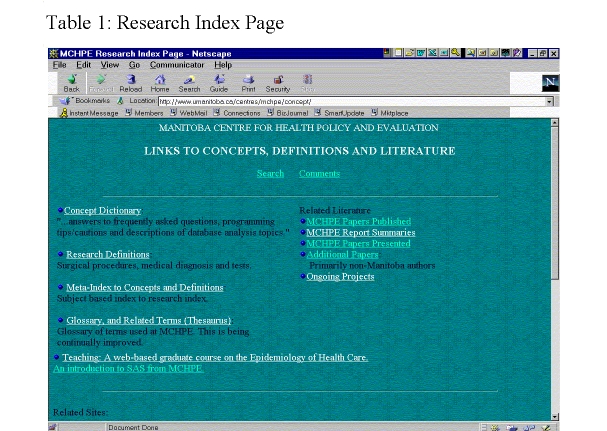
Research Index Page

Four main headings (under the Concepts/Research button on the home page) organize information in the external website: Concept Dictionary, Research Definitions, Meta-Index to Concepts and Definitions, and Glossary and Related Terms (Thesaurus). Two other headings, Teaching and Related Literature, are designed to help researchers. Cross-links facilitate information retrieval. Figure 11 illustrates how the information available to external users is organized.

The Concept Dictionary provides one (or more) operational definitions of concepts used by health researchers, and describes in detail the development of new variables or creation of variables based on existing data (e.g., age from birth year; Full Time Equivalent (FTE) Physicians from physician billing numbers, billing location, individual patient visitation). The creation of variables requiring a complex set of steps is also outlined. The content of the Concept Dictionary, like that of the Data Dictionaries (see below), was designed to provide the information necessary for research continuity.

**Figure 12:**
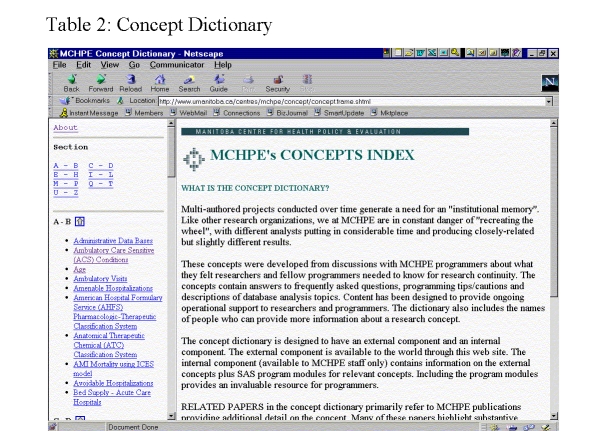
Concept Dictionary

Figure 12 presents a sample of the terms included in the Concept Dictionary. To illustrate, the entry under "Ambulatory Care Sensitive Conditions" defines the concept, cites relevant research from which the concept was originally defined, and directs those seeking additional information to the MCHPE programmer or researcher most familiar with the concept. The entry under "Age" describes various methods for calculating age and format requirements. Entries often include cautions associated with the concept, as well as possible remedies. For example, the write-up for calculating the exact age for a particular date warns that Manitoba physician claims do not generally contain the "date of birth" field and suggests how this information can be acquired.

Some concept definitions call for a more lengthy discussion of measurement. Various issues associated with the Manitoba database are outlined, including the following: standard data exclusions, strategies for avoiding double-counting of hospital visits over multiple years and concurrent stays, and definitions of geographical regions. The entry "Health Status Indicators" includes a section describing six indicators, a section of related definitions, a section noting key references, and a table listing various indicators and their associated ICD-9-CM codes.

**Figure 13:**
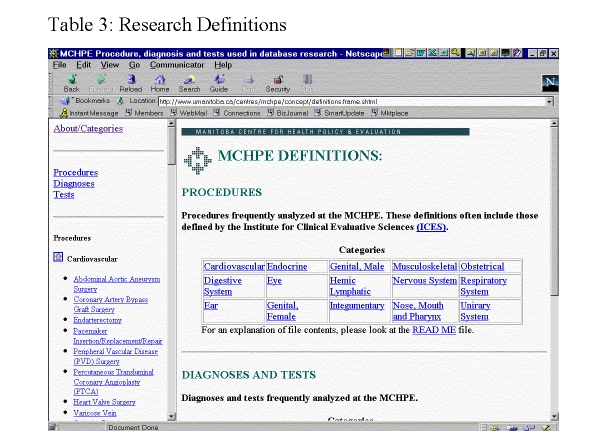
Research Definitions

The Concept Dictionary is organized alphabetically with numerous cross-references to facilitate logical connections among various dictionary entries. For example, MCHPE plans to develop linkages from concepts such as "income group calculation" to specific variables (e.g., municipal and postal codes) in the data dictionary from which it was derived. The "Research Definitions" heading (Figure 13) defines the codes for common surgical procedures, tests, and diagnoses derived from classification systems such as ICD-9-CM and physician tariff codes, and from common groupers such as DRG, RDRG, and CMG. Where relevant, cross linkages are made to Concept Dictionary entries.

**Figure 2:**
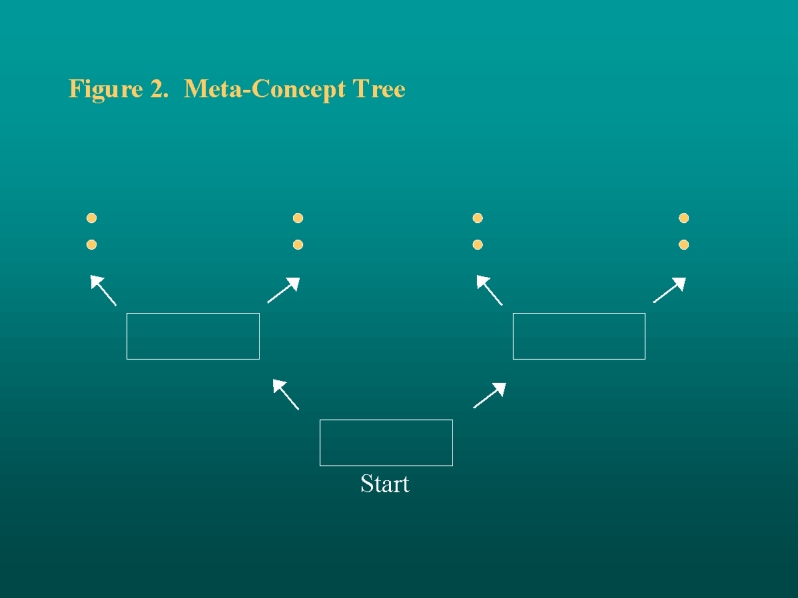
Meta-Concept Tree

The Meta-Index to Concepts and Definitions is designed to make information retrieval easier. The Meta-Index organizes concepts and definitions according to the Medical Sub-Heading (MeSH) system developed by the National Library of Medicine. MeSH allows the researcher to start with a broad category of interest and increase the specificity by choosing pertinent "branches" of the Meta-Concept "tree" (Figure 2). Although MeSH does not currently contain any content regarding public health, necessary modifications were kept to a minimum to adhere as closely to the original standard as possible.

**Figure 14:**
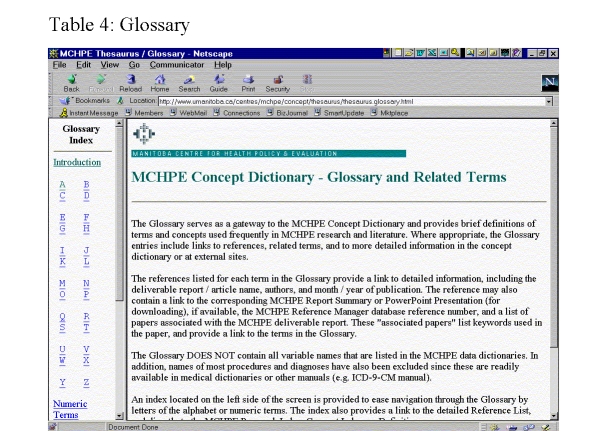
Glossary

A Glossary of terms facilitates access to information by a broader audience. The range of research terms and abbreviations can make navigation through areas of the knowledge repository unwieldy. More than one thousand terms from MCHPE government deliverables and research papers were generated as possible entries. The Glossary is organized alphabetically and designed as a quick reference to possibly unfamiliar terms encountered while reading MCHPE work. Currently, the content under each entry in the Glossary includes a brief definition (as many as are relevant), a list of synonyms and related terms, a reference list for papers that use the concept, and, for more in-depth information, links to relevant parts of the Concept Dictionary (Figure 14).

Teaching presents the web-based version of a graduate course, "The Epidemiology of Health Care."" Given the typical course audience - a substantial number of auditors and a small number of enrolled graduate students - most of the weekly readings use links to material already available on the web: project summaries, newsletters, and entries in the Concept Dictionary. Many of the academic papers distributed in paper form are from MCHPE's June 1999 supplement to Medical Care. The Glossary incorporates links from the definition of terms to MCHPE deliverables and research papers where they appear. For teaching purposes, links that go in the other direction allow the student to produce a list of key concepts and terms associated with a given research paper or report. The course and other teaching materials (including a lecture in the Epidemiology Supercourse) are included under the Education/Resources button on the home page. Finally, the heading Related Literature includes: MCHPE Papers Published (with accompanying abstracts for recent years), MCHPE Report Summaries, MCHPE Papers Presented, Additional Papers (primarily non-Manitoba authors), and Ongoing Projects using the research definition(s).

Links to associated pages (both locally and elsewhere on the Internet) allow quick cross-references and navigation through complex topics. The search engine (Excite) allows users to search the Research Index using key words or phrases. Conversion of all documents available at the MCHPE website to HTML format, together with the addition of meta-tags with key words, has provided faster, more reliable searches. The Concept Dictionary has also been added to many general external search engines (Lycos, Hotbot, and Excite) to facilitate access.

### Internal Website

Material that is restricted in nature or irrelevant to external users is available on MCHPE's internal website. Some concepts include SAS programming, and incorporate confidential information such as hospital identifier. In other cases, exclusion from the public website is based on the proprietary nature of the code or database structure. Specific file names or locations provide structural information regarding our computer systems; external users are also excluded from this information. Finally, use of the internal dictionaries assumes familiarity with MCHPE data organization; variable names meaningful to only MCHPE staff would not be useful on the external web site.

Data Dictionaries are highly valued among information systems users; they typically list and describe variables and the structure of specific files of any given set of databases. MCHPE has separate data dictionaries for each major set of data listed in Figure 1(e.g., hospital, medical, nursing home, provider, vital statistics, cost, pharmaceuticals). These data dictionaries are routinely stored as part of the various MCHPE data files. The contents include standard names for variables, formats, interpretation of the variables, and an overview of known problems and recommendations. Pages are designed to facilitate access to database research concepts and definitions. Given the confidential nature of the information, the data dictionaries are not currently available on the external Web pages.

### Quality and Bias

Notwithstanding the wider applicability of MCHPE-produced work, our knowledge repository has emphasized ideas and methods associated with one research center. The risk of bias through presenting a single viewpoint needs to be addressed, particularly as the Concept Dictionary expands and perhaps becomes a "destination site" for health care researchers using administrative data. Unlike peer-reviewed papers, the content of web sites generally is not filtered through a critical academic review process. Online knowledge systems must develop and incorporate methods for ensuring information quality. Our procedures to address this issue might help others:

1. Develop clear standards and procedures for determining the content of the knowledge system. Clearly, quality is related to the process by which information is added; some items in the Concept Dictionary are identified as coming from papers and reports written elsewhere. Concepts originating in Manitoba must be tested with MCHPE data, for example, before being entered in the Concept Dictionary. The principal researcher or programmer typically works with an experienced research assistant to write entries for the Concept Dictionary as part of the documentation process for any research project or publication. Additionally, concepts are added using a style which notes:
  - Name of concept and date of information presented on the page.
  - A basic description, including both situations where the concept is relevant and limitations or cautions on use of the concept.
  - SAS code (or location of such code) and/or SAS macros used to create the concept [6]. (At MCHPE almost all programming is performed using SAS. When other programming languages are used, the appropriate code is provided).
  - SAS formats and necessary labels (or their location).
  - Other information such as relevant contact(s) and any published papers using the concept.
2. Develop procedures to keep the knowledge system current and accurate. MCHPE is considering a position for a "knowledge expert," an individual with substantive knowledge of the databases and research concepts who can develop quality-control procedures.
3. Solicit feedback. Other users of the knowledge base may supplement the information provided or define a concept differently. At MCHPE, we are seeking feedback via an entry on the Concept Dictionary home page. Other possibilities for broadening users' perspectives include incorporating information on new concepts and alternative definitions, and increasing the number of links to other research centers.
4. Make evaluation an integral part of development and maintenance. Currently, MCHPE is documenting who is using different parts of the web site.
5. Be explicit. The process of making information explicit permits individuals to evaluate, use, or develop it in ways that ultimately advance the research process.

## Results

**Figure 3:**
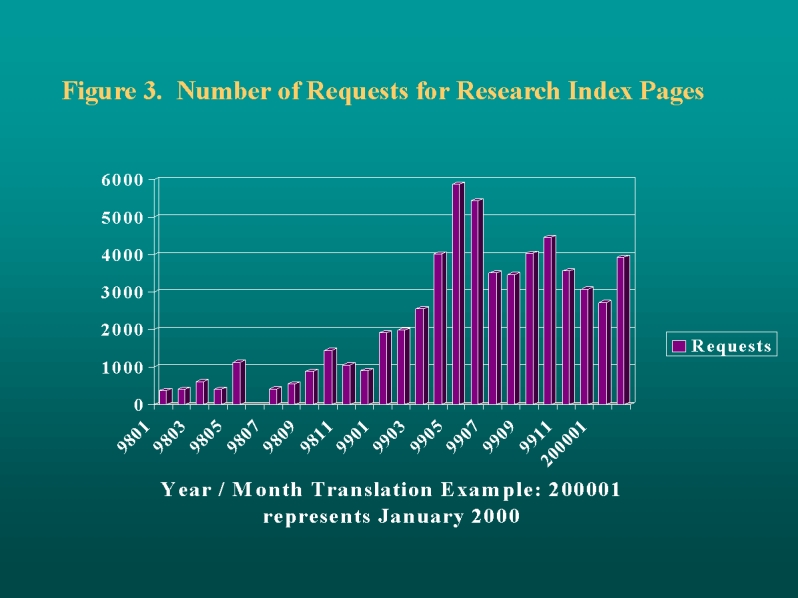
Number of Requests for Research Index Pages

**Figure 4:**
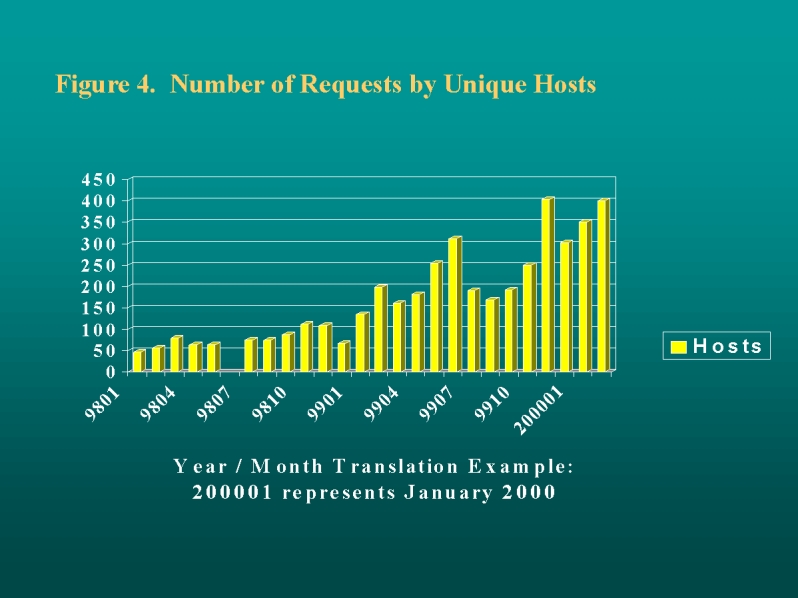
Number of Requests by Unique Hosts

The MCHPE website continues to reach an increasing number of national and international health administrators, researchers, and students. Reports, report summaries, and newsletters are available from the home page. In May 1999, MCHPE received close to 16,000 hits on the website's pages; almost 6,000 of these used the knowledge repository. Web traffic grew at an average rate of over 35% each month during the first part of 1999 (Figure 3 and Figure 4). Since then, traffic on the knowledge repository has dropped to around 4,000 page hits per month while the number of unique hosts has climbed to around 400. Depending upon whether or not a course is scheduled, between 1,000 and 3,000 hits per month have been recorded on the Education pages. When new reports are issued on the MCHPE web site, traffic generally increases in the following month. The listing of the MCHPE lecture "Studying Health and Health Care" on the Epidemiology Supercourse's 24 mirrored servers around the world will increase impact of the work, but complicate measurement of page hits [7]. Finally, we believe the internal pages are being heavily used by MCHPE staff; such usage is currently being measured.

Several national and international projects have relied on the knowledge repository fairly extensively. A large cross-national project based at Stanford University has used an index described in the Concept Dictionary (the Charlson Comorbidity Index) to help control for comorbidity before cardiovascular surgery. In another project, researchers in a five-province study funded by the Canadian Population Health Initiative will be operationalizing Continuity of Care as described in the Concept Dictionary

## Discussion

### Benefits of a Knowledge Repository

Organizing and structuring information into a knowledge repository can:

1. Promote standardization: An online information system encourages the use of standard terminology and methodology. The Concept Dictionary helps make explicit the assumptions associated with the development of a measure.
2. Increase efficiency: New projects at MCHPE often require complex programming and conceptual development. Our approach allows this intellectual property to be placed in the collective memory of MCHPE. Appropriate documentation, including descriptions of measures and detailed programming code, prevents duplication and protects the substantial time investment that such work involves. This process also keeps researchers current regarding concepts that may be modified from one project to the next.
3. Capture implicit knowledge: Capturing and making available tacit knowledge is a challenge for all organizations [5]. Frequently, a very small number of people know a great deal about the data or a specific concept. Such institutional knowledge needs to be saved in a convenient format to avoid missing important details and generating unnecessary effort to track down the expert on a specific topic. Making implicit knowledge explicit can lead to the discovery of a novel approach, or the identification of critical shortcomings in ongoing or completed projects.
4. Facilitate collaboration: Project-based websites, a recent addition to the online information system, can facilitate collaborations between MCHPE investigators and researchers from other centers. A website created for a three-province study, for example, lists the investigators from each of the provinces involved and provides a "What's New" section of project website updates. Overviews of each of the component studies include detailed methodology descriptions of data sources and eligibility criteria, as well as key concept definitions and analytic methods specific to the study. Relevant links to the Concept Dictionary and to external websites (such as the Canadian Medical Research Council and National Health Research and Development Program) provide users with additional project information.
5. Aid project management: Multi-site projects can obviously benefit from the use of web pages to coordinate the flow of information in a timely fashion. Operationalizing concepts using administrative data from different political jurisdictions is complicated by factors such as:
  - Variations in data set characteristics and coding conventions. Not only do the contents of the data sets differ, the meaning underlying apparently similar variables may also differ among provincial data sets.
  - Varying demographics. An apparently straightforward decision in one setting may be complicated in another. For example, rapid suburban growth in Calgary, Alberta has made it necessary to interpolate between old census figures and projections from the latest results to define urban Calgary. In contrast, census data that are several years old present no special problems for estimating income quintiles in slow growth Manitoba.
  - Deal with conflicts between building on previous research and developing new approaches to resolve methodological problems: since the boundaries of Statistics Canada's enumeration areas do not always correspond with postal codes, appropriate census values for neighborhood income and educational statistics must be assigned to postal codes overlapping two or more enumeration areas. For the Manitoba postal codes, MCHPE researchers have assigned average income and education using population-weighted means from the overlapping enumeration areas. This procedure was utilized for 1986, 1991, and 1996 census data and designed to facilitate longitudinal research. Starting with 1996 census, Statistics Canada developed a different, probabilistic method of converting between enumeration areas and postal codes allowing a more consistent, repeatable method of assigning income and education values to individuals within these postal codes [8]. Results based on this methodology differed very slightly from those generated using population-weighted means, but nonetheless complicate cross-sectional and longitudinal comparisons.
6. Disseminate information: The MCHPE web-based system facilitates dissemination of current information to a wide range of users. The Glossary, in particular, is directed toward researchers who may vary significantly in their familiarity with technical terms. Links to other terms, combined with information about key "contacts," permit access to details not normally published in peer-reviewed articles. Income quintiles, for example, have been widely described in journal articles, but the specific methods for generating them have not been published [9]. The "Ongoing Projects" section permits users to determine (without waiting for publication) the current status of various projects undertaken by MCHPE researchers.

### Future Directions

The knowledge repository may be characterized as a tool for increasing the flow of high quality information relevant to health services research. Meeting our objectives requires developing this resource in two directions: First, honing technical features will allow users who vary considerable in their expertise to more easily navigate through the available information. Second, the content must be continually updated and expanded to reflect the activities at MCHPE and related centers.

Considerable effort is being directed toward enhancing the number of links within dictionaries, from dictionaries to other sections of the MCHPE web site, and to non-MCHPE sites (click, for example, on PHRU Concept Dictionary; this was developed by the Population Health Research Unit at Dalhousie University). Extensive links among various glossary terms have recently been established (see, for example, A in the Glossary Index). We also hope to provide more direct access to concepts and definitions associated with individual papers by means of a search engine interface that returns all links (concepts, definitions and terms) used in a specific paper or report.

The content of the repository will expand as we incorporate new topics and new directions within MCHPE. Enhancing the linkages between geography and information (GIS) is an important direction for both research and dissemination. As MCHPE databases increase to include social service and educational information, terms and methods relevant to these new areas will be incorporated. We plan to include as much high-quality information as possible in our external web pages, while providing researchers with an informed sense of the issues involved in working across jurisdictions. Given our funding by Health Canada and various federal and provincial agencies, other researchers and organizations are permitted to copy parts of the dictionary. An advisory committee will generate suggestions for future additions, with considerable attention being given to what is most useful beyond Manitoba. Finally, we need to constantly address the tradeoff between adding content and organizing the information. Monitoring use of different entry points (Concept Dictionary, Research Definitions, Meta-Index, etc.) to the substantive material should help our investment decisions; considerable activity has been associated with each entry point.

Our efforts have been directed toward making documentation in the Research Index a routine component in project planning and development. The WWW, with its multi-dimensional capabilities for hot links and structured documentation, has provided a vehicle for meeting such objectives. As a knowledge base, the MCHPE online resource is new and possibly unique to health services research. We have only scratched the surface in terms of this resource's potential for training and education - locally, nationally, and internationally. The graduate course on the web site supported distance education in the fall of 1999. Visitors to MCHPE have already found the knowledge repository helpful in reviewing material before arrival.

Our experience is encouraging since accessibility to information, together with opportunities to receive feedback, moves us closer to an interactive format where research ideas can advance rapidly through an iterative process. With safeguards to assure information quality and increased opportunities for collaboration, we anticipate some real innovations in how health services research and epidemiology are conducted.
